# Evaluation of the CNC^®^ prosthetic system in recurrent breast cancer patients with chemotherapy-induced alopecia: a pilot study

**DOI:** 10.1186/s12905-022-02080-7

**Published:** 2022-12-03

**Authors:** Alessandra Petruzzi, Anna Maria Mancuso, Sara Alfieri, Antonella Esposito, Gabriele Infante, Rosalba Miceli, Stefano Ospitali, Carla Ida Ripamonti, Claudia Borreani

**Affiliations:** 1 Salute Donna Onlus Association, Milan, Italy; 2 Clinical Psychology Unit, Fondazione IRCCS Istituto Nazionale dei Tumori, Via Giacomo Venezian 1, 20133 Milan, Italy; 3 Laboratory of Medical Statistics, Biometry and Epidemiology “G. A. Maccacaro”, Department of Clinical Sciences and Community Health, University of Milan, Milan, Italy; 4 S.S. Biostatistics for Clinical Research, Department Epidemiology and Data Science Department of Applied Research and Technological Development, Fondazione IRCCS Istituto Nazionale dei Tumori, Milan, Italy; 5 Advihair Srl, Zola Predosa, Bologna, Italy; 6 Supportive Care in Cancer Unit, Fondazione IRCCS Istituto Nazionale dei Tumori, Milan, Italy

**Keywords:** Body image, Chemotherapy-induced alopecia, CNC^®^ device, Mixed-method, Quality of life, Wellbeing, Recurrent breast cancer

## Abstract

**Background:**

Chemotherapy-induced alopecia (CIA), although generally reversible, is felt as extremely distressing by patients with breast cancer. A certified medical device (Capelli Naturali a Contatto^®^—CNC^®^) was produced to provide patients with a personalized scalp prosthesis, reproducing the patient’s original hair, resistant to any type of everyday or sporting activity, and hairdressing.

**Aims:**

The present study aimed to evaluate the impact of the CNC^®^ device on the patient’s perception of their body image, psychological wellbeing, satisfaction, strengths and weakness of the CNC^®^ device.

**Method:**

A pilot study was carried out on 21 patients affected by CIA due to recurrent breast cancer. A mixed quantitative/qualitative method was used, including administering a questionnaire and a focus group.

**Results:**

Based on the Body Image Scale, body image perception improved after 3 and 6 months using the device in the 20 patients who answered the questionnaire. No significant change over time emerged for the six dimensions investigated by the Italian version of the Psychological Well-Being Scale. The thematic analysis of the focus groups showed six themes: definition of the prosthetic device, acceptance of the proposal, experience with the conventional wig, strengths, weaknesses, economic issues.

**Conclusion:**

Compared to the previous experience of CIA and the standard wig, the use of the CNC^®^ device improved everyday life and may be proposed to women undergoing chemotherapy and expecting alopecia to prevent discomfort, social embarrassment, and compromised body image.

**Supplementary Information:**

The online version contains supplementary material available at 10.1186/s12905-022-02080-7.

## Background

Breast cancer is the most common cancer among women in developed countries and presents several disabling complications related to treatment [1]. Alopecia is a common side effect of chemotherapies used to treat breast cancer [2]. The proportion of patients receiving cytotoxic agents who experience alopecia was as high as 65%. Although generally reversible, alopecia is ranked among the most troublesome adverse events, being described as distressing and affecting body image [3, 4]. The timing and seriousness of chemotherapy-induced alopecia (CIA) depend on the drug and treatment schedule [2, 5, 6]. Alopecia, as stated by Zannini et al. in a qualitative study, is experienced as a traumatic event that challenges a woman’s femininity. In this experience, the wig was perceived as helpful, only within a specific supporting program [7]. Changes in body image during CIA have long been described [8]. It was demonstrated that body image worsened during chemotherapy but did not return to normal or improve when patients experienced hair regrowth. In addition, the CIA was found to negatively affect Quality of Life (QoL) associated with a relevant psychological burden [9, 10]. Indeed, body change was among the factors predicting lower QoL scores in breast cancer survivors [1]. In addition, changes in body image perception impact self-efficacy to cope with cancer, as coping self-efficacy is linked to well-being through multiple cognitive, emotional, and behavioral pathways [11].

Some strategies, such as scalp cooling, topical treatment with vasoconstrictors, minoxidil or the prostaglandin analog bimatoprost, and topical or systemic calcitriol, have been introduced during the last decades to prevent CIA in breast cancer patients. Still, all these interventions had reduced activity and several tolerability issues [9, 12, 13]. Camouflage with wigs is a very common approach but may not satisfy all patients’ needs. Indeed, a qualitative study on women with breast cancer in Sicily (an Italian region) found that the wig was helpful given that an aesthetic/care wig program is provided to improve coping abilities [7].

Helping women manage this problem may improve the perception of body image and facilitate the everyday life of the patient and her surrounding friends [2, 5, 6].

With the objective to have an efficient and satisfactory camouflage, the “Capelli Naturali a Contatto^®^” (CNC^®^) device was produced by Advihair srl (Zola Predosa, Bologna, Italy). It aims to provide patients with a personalized scalp prosthesis, reproducing their original hair, resistance to any type of everyday or sporting activity, and hairdressing. This device is expected to improve the perception of body image and psychological well-being during chemotherapy by reducing the impact of alopecia.

The present study aimed to evaluate the impact of the CNC^®^ device on the patient’s perception of their body image, psychological wellbeing, satisfaction, strengths and weakness of the CNC^®^ device. This pilot study was carried out on female patients affected by CIA due to recurrent breast cancer.

## Methods

### The prosthetic device

The CNC^®^ prosthesis is a certified (ISO 9001–14000–18000) medical device produced by a patented process by Advihair srl. Briefly, a polymer, manufactured in-house by a proprietary method, is shaped on the mold of the patient’s head and colored with a biologic pigment according to her scalp skin color. Natural untreated hair with the features of the patient’s hair is applied by hand on the molded polymer. Specialized personnel fit the device to the head and fix it with an adhesive substance; a patch test rules out possible intolerance. The device needs sanitization once a month. A total of 21 devices were donated by Cesare Ragazzi Laboratories (Italy) to perform this study.

Patients enrolled in the study were provided with a personalized device. They wore it as long as desired and they were allowed to remove the device when satisfied with the length of hair. Application began before alopecia was induced by chemotherapy.

### Participants

Enrollment was performed from October 2017 to July 2020 within the Complex Structure of Medical Oncology 1 of Fondazione IRCCS Istituto Nazionale dei Tumori of Milan. Patients with recurrent breast cancer and CIA, and meeting the following inclusion criteria were enrolled: use of a traditional wig in the previous experience of CIA, age > 18 years, absence of cognitive disorders, anxiety and/or depression, and willingness to apply the proposed device. A psychologist [AP] explained the study aims and procedures to the patients. Thereafter, if willing to use the prosthetic system and participate in the study, they signed a written consent form.

The patient sample was limited to 21 subjects due to the number of available devices.

### Endpoints

The primary endpoint was to evaluate the impact of wearing the CNC^®^ prosthetic system in patients with a relapse of breast cancer with CIA on body image and psychological well-being. The secondary endpoints included satisfaction, strengths and weaknesses of the device. Both endpoints were compared with the previous use of a “conventional wig.”

### Assessment

A mixed-method approach [14] which includes a survey and a focus group was used to achieve research aims.

*Survey* A questionnaire was administered to all participants.

The Italian validated version of the Body Image Scale (BIS) was used to evaluate the perception of body image [15, 16]. This 10-item scale investigates affective, behavioral, and cognitive dimensions of body image in patients affected with neoplasia. The total score ranged from 0 to 30: zero scores represent no symptoms or distress; higher scores correspond to increasing symptoms and distress or more body image concerns.

The psychological well-being was assessed by the Italian version of the Psychological Well-Being Scale (PWB) [17, 18]. Answers are given on a 6-point Likert scale, and 18 items are considered. This questionnaire investigates six dimensions: autonomy, relationships, mastery of the environment, personal development, acceptance of oneself and purpose in life. Each dimension ranged from 3 to 18: the higher the score, the higher the psychological well-being.

The perception of hair was evaluated by two additional items *(“Hair is important to me”* and *“Hair is an important aspect of my look”*) concerning how important hair is to the patient and the impact on physical appearance. Each answer is given on a 5-point Likert scale, and the resulting score was considered their average: the higher the score, the higher the importance of hair.

Sociodemographic and clinical data of patients were collected. Occurrence of any discomfort with the device was also recorded.

The beginning of a new regimen of chemotherapy was the baseline. At this time, patients were required to answer the BIS by referring to their previous experience with a conventional wig, while the PWB investigated their current well-being. Assessments were repeated 3 and 6 months after applying the device, asking the patient to evaluate the recent experience with both BIS and PWB.

*Focus group* The first 12 patients who completed the use of the device were asked to participate in the focus group. A focus group is a qualitative research technique used to deepen a theme through group interaction of people who have a common experience or characteristic [19]. A psychologist with experience in qualitative research [SA] facilitated the focus group. A semi-structured interview was used for the discussion, and the interviewer used a non-directive modality. The following topics were considered: baseline expectations regarding the device, comparison of standard wigs with the CNC^®^ device, perception and evaluation of the device, possible weaknesses and willingness to buy the device.

### Statistical analysis

The scores of the perception of hair, BIS and PWB scales were calculated according to the developer’s indications. Descriptive statistics were applied to obtain summary results for each score by time point.

Nonparametric rank-based models for longitudinal data were used to model the time trends for each scale [20, 21]. Those models are suitable for non-Gaussian-distributed variables, are robust to outliers, and exhibit competitive performance for small sample sizes. These models allow to statistically test the null hypothesis of no time effect using an ANOVA-type test [22, 23]. As additional analysis when ANOVA-type test was significant, the Wilcoxon–Mann–Whitney exact paired test adjusted for tied values was used to compare distributions of scores at baseline versus 3 months and baseline versus 6 months [24].

The statistical significance level of tests was 0.05. Analysis was conducted in R [25].

Cronbach alpha values were calculated at baseline (complete data), with Bootstrap 95% CI with 2000 replications.

### Qualitative analysis

The focus group was audio-recorded, and transcriptions were analyzed through a paper–pencil thematic analysis [26]. Thematic analysis is one of the most popular method of analyzing qualitative data. With the word “theme” we referred to a topic or concept that emerges throughout the text and that answer to the research aims. The themes can be articulated into sub-themes, each of them focused on specific aspects. The qualitative study followed the Consolidated criteria for reporting qualitative research (COREQ) [27].

## Results

### The results of the questionnaires

*Participants *A total of 21 patients were assessed at baseline. Table 1 reports the sociodemographic and clinical characteristics, while Table 2 shows the chemotherapeutic regimens at baseline. After 3 months, one patient was not assessed because of a declining clinical condition. After 6 months, four patients were not evaluated because of unavailability to assessment, disease progression, death or early removal of the device after regrowth of hair. So, the analysis sample was restricted to 20 patients observed at least twice and 16 patients 6 months after the application of the device. Patients wore the device for a mean time of 9 months (range: 4–15 months). In Supplementary material, the distributions of scales which have a non statistically significant difference across time were showed.

**Table 1:** Sociodemographic and clinical characteristics of patients

Sociodemographic and clinical characteristics	*n* = 21, *n*
Age (years), mean (range)	60.24 (44–73)
*Degree*	
Elementary education	2
Level 12	4
High school	9
Bachelor’s degree	6
*Marital status*	
Married	19
Widow	2
Disease duration (months), mean (range)	169.05 (30–276)
*Treatment at baseline*	
Chemotherapy	20
Chemotherapy + radiotherapy	1
*Cancer stage*	
Recurrence	21

**Table 2:** Chemotherapy received by patients at baseline

Patients	Drugs
1	Nab-paclitaxel
2	Paclitaxel
3	Abraxane
4	Carboplatin + paclitaxel + trastuzmab
5	Eribulin
6	Nab-paclitaxel
7	Nab-paclitaxel
8	Taxol
9	Eribulin
10	Eribulin
11	Paclitaxel + pertuzmab + trastuzumab
12	Liposomial doxorubicin
13	Paclitaxel
14	Paclitaxel
15	Paclitaxel
16	Carboplatin + paclitaxel
17	Nab-paclitaxel + trastuzmab
18	Nab-paclitaxel
19	Epirubicin
20	Taxotere + cyclophosphamide
21	Carboplatin + paclitaxel + trastuzmab

**Fig. 1:**
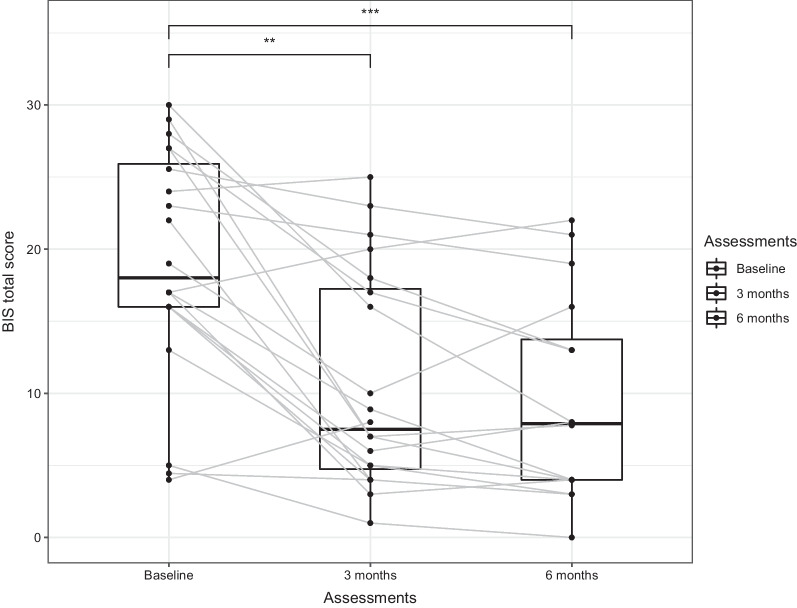
Distributions and individual trends of total mean scores of Body Image Scale (BIS) at baseline (*n* = 20), after 3 months (*n* = 20), and after 6 months (*n* = 16) of device use. Notes: (**) *p* < 0.005 3 months versus baseline and (***) *p* < 0.001 6 months versus baseline, Wilcoxon–Mann–Whitney test

*Body image perception and psychological well being *The total mean score of the BIS items was 19 (range 4–30) at baseline and reduced to 10.6 (range 1–25) after 3 months. It further reduced to 9.4 (range 0–22) at month 6. The difference at month 6 versus baseline was statistically significant with ANOVA (p < 0.001) and the Wilcoxon–Mann–Whitney test (*p* < 0.005 at 3 months, and *p* < 0.001 at 6 months), showing a persistent improvement of body image perception (Table 3, Fig. 1). At baseline, Cronbach alpha value for BIS was 0.90 (95% CI 0.76–0.95), showing a good scale’s reliability.

**Table 3:** Mean scores (range) of perception of hair relevance, Body Image Scale and Psychological Well-Being scales at baseline, months 3 and 6 after application of the device

	Baseline (*n* = 20)	3 months (*n* = 20)	6 months (*n* = 16)	Time effect ANOVA-type (*p*)
Perception of hair relevance	4.2 (4–5)	4.2 (2–5)	4.1 (3–5)	0.327
BIS total score	19 (4–30)	10.6 (1–25)	9.4 (0–22)	< 0.001
PWB: autonomy	11.9 (7–17)	10.8 (7–15)	10.6 (7–14)	0.132
PWB: environmental control	11.9 (8–15)	11.7 (9–14)	12.3 (10–15)	0.413
PWB: personal growth	12.6 (9–16)	12.2 (9–16)	12.2 (8–17)	0.633
PWB: positive relationships	12.8 (7–16)	12.2 (7–16)	12.4 (9–17)	0.429
PWB: aim in life	10 (6–13)	9.1 (5–13)	9.2 (5–12)	0.077
PWB: self-acceptance	11.6 (8–15)	12.3 (9–17)	12.2 (8–15)	0.28

*BIS* body image scale; *PWB* psychological well-being

On the contrary, the scores related to other scales (perception of hair relevance, and the six PWB subscales—autonomy, environmental control, personal growth, positive relationships, aim in life, self-acceptance) were not significantly changed over time (Table 3; Additional file 1: Figs. S1–7). These scales, each one with 2 or 3 items, proved not to be reliable (perception: alpha = 0.6, 95% CI: 0.3–0.8; autonomy: alpha = 0.09, 95% CI − 1.5–0.6; environmental control: alpha = 0.005, 95% CI − 1.0–0.6; personal growth: alpha = − 0.3, 95% CI − 1.6–0.3; positive relationships: alpha = 0.3, 95% CI − 1.0–0.7; purpose in life: alpha = 0.04, 95% CI − 1.4–0.5; self-acceptance: alpha = − 1.8, 95% CI − 7.0–0.1).

### ***The results of the focus group: the satisfaction, strengths and weakness of the CNC***^***®***^*** device***

Five patients attended the focus group out of the 12 ones who were invited. Seven out of 12 patients could not participate in the focus group because of work obligations, health issues, and difficulty reaching the hospital from home.

The analysis of the focus groups showed six themes: definition of the prosthetic device, acceptance of the proposal, experience with the conventional wig, strengths, weaknesses, economic issues. Some themes were articulated into subthemes.

1. *Definition of the prosthetic device:* When invited to define the device, the patients expressed their satisfaction, and their quotations included not only material description but also affective evaluations, mainly related to a favorable comparison with the traditional wig. A woman said: *"Wonderful. Really, the wig is a far, far ancestor of this one… because, once you put it on [the new device], with these special adhesives, it is fixed, and nobody can see it is not your hair".*
2. *Acceptance of the proposal:* All the patients declared that they were excited to accept the proposal and had been attracted by fear to wear a wig again: *"It was a new idea, and I accepted immediately, without a second thought, because I was willing to do anything to avoid the wig."*
3. *Experience with the conventional wig:* Quotations about the experience with traditional wigs were unfavorable due to several issues: wigs look unnatural, can move while the person does not realize it, are to be removed at night so that the person feels bare next to her partner. A woman said: *“A wig is a separate object; it does not become a thing that is a part of what one is….The last time I wore a wig, I fell, and the wig flew, turned, and moved…”.* Another one: *“When I first had it, my husband never saw me without the wig; I took it off when it was dark, and I put on the turban because I did not want to be seen by anybody.”*
4. *Strengths*: Patients expressed their satisfaction with the device itself but also for the professional assistance received. So, this theme was articulated into two subthemes, (a) the device and (b) the assistance. The first one pertains to the device characteristics. The device helped the patients not to “feel ill” because it looked like natural hair, as this patient’s words express: “*One does not see oneself ill…the important thing of this device is that it makes you feel less ill. A hard side of this disease is that it is not a headache that goes, and you are well again; it is something you wake up with every morning…If you think about it every morning, you go, jump out of the window, and it is finished… But your lifestyle must be that ‘you put it aside’*.” In addition, when the management of everyday life and QOL were discussed, some device features were described as important: you ‘do not see’ the device, the device did not move on the scalp, had good quality hair, and was easily managed. As an example, one patient explained that she had been able to live a normal life with the device: *“I went out as usual, while I had great problems before.”* A woman described a feeling of comfort and ease: *“You can take a bath, have a walk, without any risk. When my nieces touched my hair, I was not afraid that it could stick to their hands”*. Finally, patients were happy to spend a short time on device maintenance.
  The second subtheme pertains to assistance. The patients reported that the personnel who prepared, applied and cared for the CNC^®^ device were friendly and professionally skilled (for example, one patient said: “*They are extraordinary*”). However, some concerns were reported, as an example: *“If you do not go and have it sanitized, it smells like cheese”* or “*It is difficult to be perfect*”.
5. *Weaknesses*: criticalities were divided into three subthemes, which were related to (a) the device, (b) the assistance, (c) feeling comfortable. The first subtheme covers several issues, among which the main and most felt one was that the device did not guarantee satisfying perspiration and induced itching, as this patient says: *“At the beginning, I had problems with itching; I could not bear it, and I had it removed”*. In addition, the device could not be removed at night (bedtime), and the adhesive was not comfortable: *“Then, I said it was enough for me because I could not sleep, I got psychologically ill because, if you do not sleep, at night, with that thing on your head…”*. The second subtheme (disagreeing with one strength previously reported) was that the hairdresser of the device producer was not very satisfactory, as this patient reports: *“I had some problems with a haircut. Well, I am particular with haircut […] every time I went there, I complained, I said I could not manage it…. The hair was thick, and I have thin hair”*. In addition, the time required to obtain the device was quite long, and several sessions were necessary for the preparation. Eventually, within the third subtheme are present two issues, not related to the device itself nor the assistance, but due to subjects’ emotional experiences. The first pertains to feeling allowed to go to a hairdresser who does not work in the device assistance. Some subjects felt guilty and felt they were betraying those who had provided the device free of charge. The second issue of the third subtheme concerns feeling uncomfortable when taking a bath in the sea or the swimming pool, as this patient reports: *“I never took a bath […] because when you get out, it is not as your hair”*.
6. *Economic issues:* all participants imagine a very high cost for the device (between 4.000 and 10.000 euros); some of them said that they were so satisfied with the device that they were willing to pay the assumed cost: *“I am willing to pay how much as needed, because I am very satisfied, and so, one can make a sacrifice.”*; others could not have afforded it.

## Discussion

We performed a pilot study to evaluate the personal experience with the CNC^®^ prosthetic device compared to standard wigs by female patients with recurrent breast cancer and with the second event of CIA. Overall, a quantitative assessment of image perception and a qualitative evaluation of the experience suggested an advantage of the new device in comparison with the standard wig. In the literature, we found that patients were pleased with the wig in that it helps them maintain their normal appearance [7, 28, 29]. Nevertheless, Versluis et al. [28] found that most patients felt a changed identity despite the use of a head covering.

The present study showed that the use of the CNC^®^ device seems to be very helpful in breast cancer patients’ process of identity restoration. More specifically, this device improves patients’ body image perception, compared to the previous experience of alopecia and the common wig. Our results suggested that the perception of body image was better during the use of the device compared to the period of wig use.

In the focus group, narratives by the patients related the use of the device with a reduced feeling of illness. Patients reported that people did not realize that their hair was not natural. Not perceiving the alopecia, the patients and those around them easily forgot the disease. Forgetting the disease, the patients felt healthy. Patients reported that feeling healthy may facilitate the management of everyday life activities and live one’s relationships without interfering with the theme of illness. So, the CNC^®^ device, thanks to its characteristics, seems eliminate the fear of losing their wig during social activities as reported by Zannini et al. [7]. Therefore such device could prevent social avoidance that is a strategy used by patients to cope with the hair loss, because of fear that others will see or treat them in a different way [4, 29].

A relevant drawback of the device reported by a few patients was the poor perspiration, mainly during bedtime, but this seemed to be overcome by the satisfaction with this efficient and friendly camouflage. In addition, based on this experience, the device may be improved to address the issue.

In light of the above, an improvement of activities and of relationships promoted by forgetting the illness may result in a better QOL. Avoiding feeling ill could improve health-related QOL, based on the results of a prospective study that found that improving health-related QOL of breast cancer survivors was associated with good general health perception [30]. Given that many societies stigmatise alopecia, and cancer patients report feeling of shame for being ill [31], maintaining a sense of normality can give the woman the opportunity to live tumor in a more private sphere and not suffer such social stigma.

We may suggest that a better perception of body image and a reduced concern with social stigma could help the patients cope with the disease and improve the tolerability of treatments. As hair loss will affect each patient and their family differently, tailored support could be associated with using the device to optimize physical and psychological well-being [10]. Our results, suggesting that the device could positively impact relationship ability and QoL, may be the basis for identifying welfare initiatives supporting the patients economically.

We acknowledge that this study had some limitations. Only one focus group was performed, and the theoretical saturation of treated themes could not be verified. The number of patients was reduced. A control group of women wearing a standard wig was lacking, and the experience with a standard wig was compared with the current use of the new device in the same subject; this provided an intra-sample consistency of evaluations but offered the bias due to different times of facts evaluated by the patients. The sample consisted of patients who had overcome their fear of the disease, different from patients on their first experience with chemotherapy. Results could be difficult to generalize to breast patients on the first chemotherapy. Further studies on a wider sample are needed to confirm our results.

In conclusion, based on this preliminary experience and waiting for further confirmation, the CNC^®^ device seems to be a reasonable proposal for women undergoing chemotherapy and expecting alopecia to prevent discomfort, social embarrassment, and compromised body image. Further investigation could explore the expectancy of patients at their first chemotherapy and evaluate the device’s impact on the caregiver.

## Supplementary Information

**Additional file1**.

## Data Availability

Results data are available upon request to the corresponding author.
