# Supplementary material for: Evaluation of the CNC® prosthetic system in recurrent breast cancer patients with chemotherapy-induced alopecia: a pilot study
Source: BMC Womens Health. 2022 Dec 3;22:492. doi: 10.1186/s12905-022-02080-7 (PMC9719124; doi:10.1186/s12905-022-02080-7)
Supplement: Supplementary file 1 — Additional file1. [file 12905_2022_2080_MOESM1_ESM.pdf]

# Supplementary material - Evaluation of the CNC® prosthetic system in recurrent breast cancer patients with chemotherapy-induced alopecia: a pilot study

Petruzzi, Mancuso, Alfieri, Esposito, Infante, Miceli, Ospitali, Ripamonti, Borreani

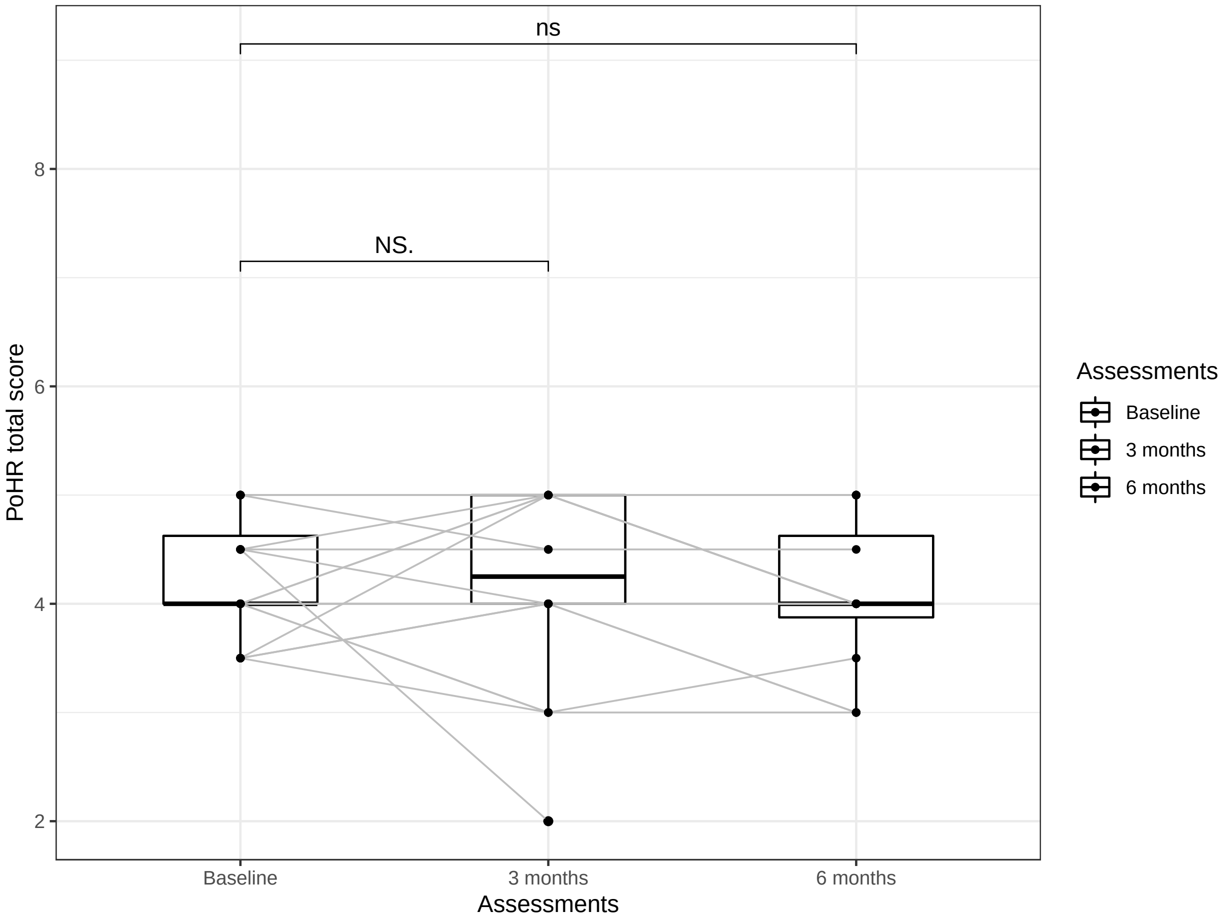


Supplementary Figure 1:
Distributions and individual trends of total mean scores of Perception of Hair Relevance (PoHR) at baseline (n=20), after 3 months (n=20), and after 6 months (n=16) of device use; (ns) not significant at Wilcoxon-Mann-Whitney test.

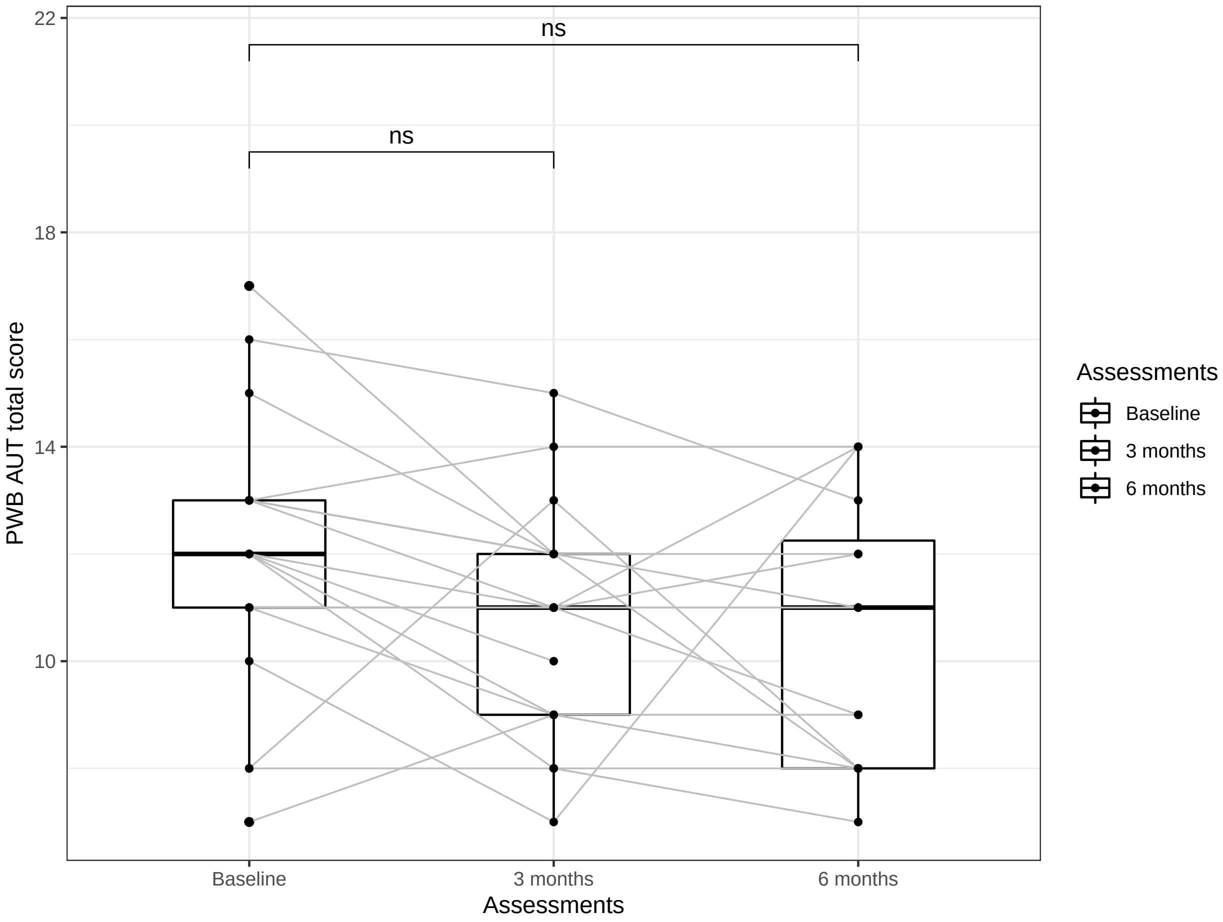


Supplementary Figure 2:
Distributions and individual trends of total mean scores of PWB Autonomy (PWB AUT) at baseline (n=20), after 3 months (n=20), and after 6 months (n=16) of device use; (ns) not significant at Wilcoxon-Mann-Whitney test.

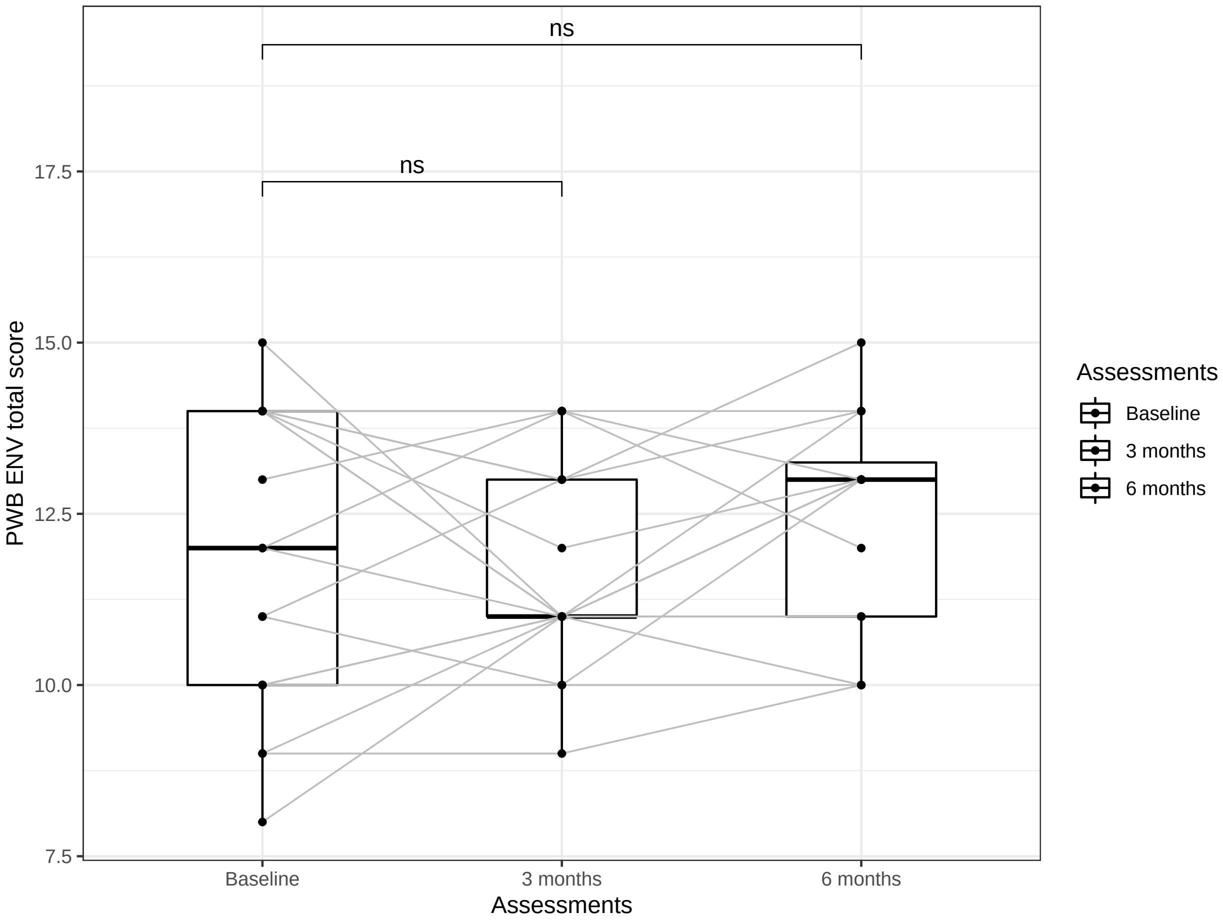


Supplementary Figure 3:
Distributions and individual trends of total mean scores of PWB Environmental Control (PWB ENV) at baseline (n=20), after 3 months (n=20), and after 6 months (n=16) of device use; (ns) not significant at Wilcoxon-Mann-Whitney test.

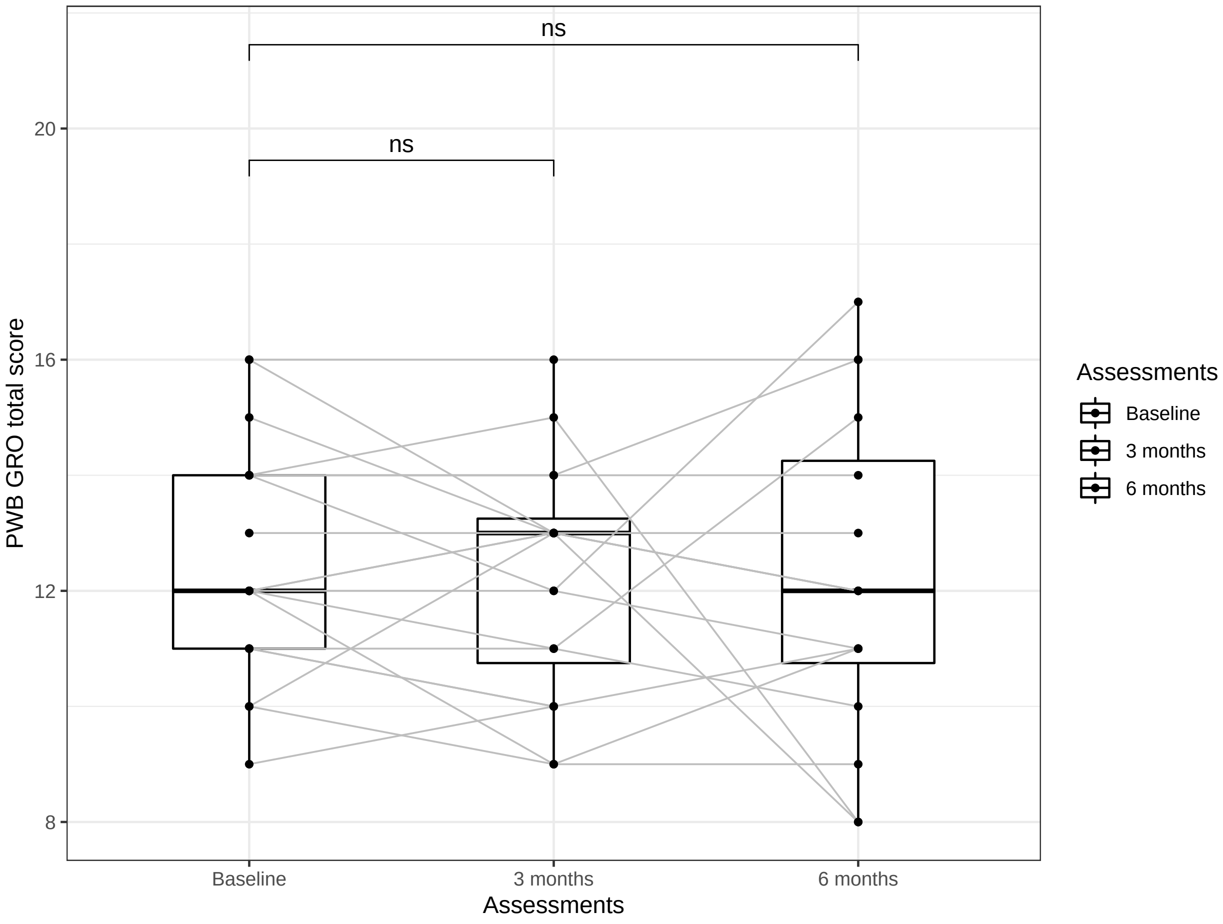


Supplementary Figure 4:
Distributions and individual trends of total mean scores of PWB Personal Growth (PWB GRO) at baseline (n=20), after 3 months (n=20), and after 6 months (n=16) of device use; (ns) not significant at Wilcoxon-Mann-Whitney test.

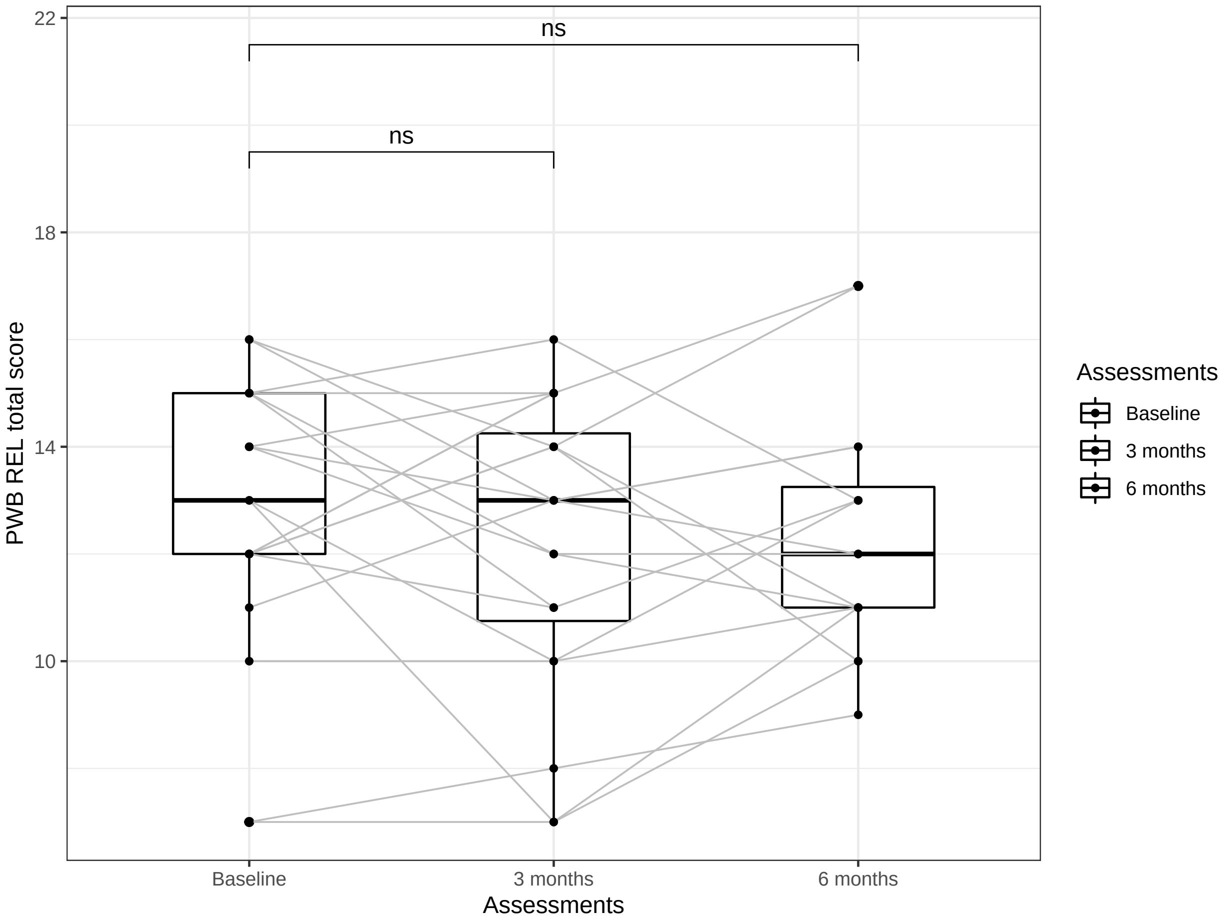


Supplementary Figure 5:
Distributions and individual trends of total mean scores of PWB Positive Relationships (PWB REL) at baseline (n=20), after 3 months (n=20), and after 6 months (n=16) of device use; (ns) not significant at Wilcoxon-Mann-Whitney test.

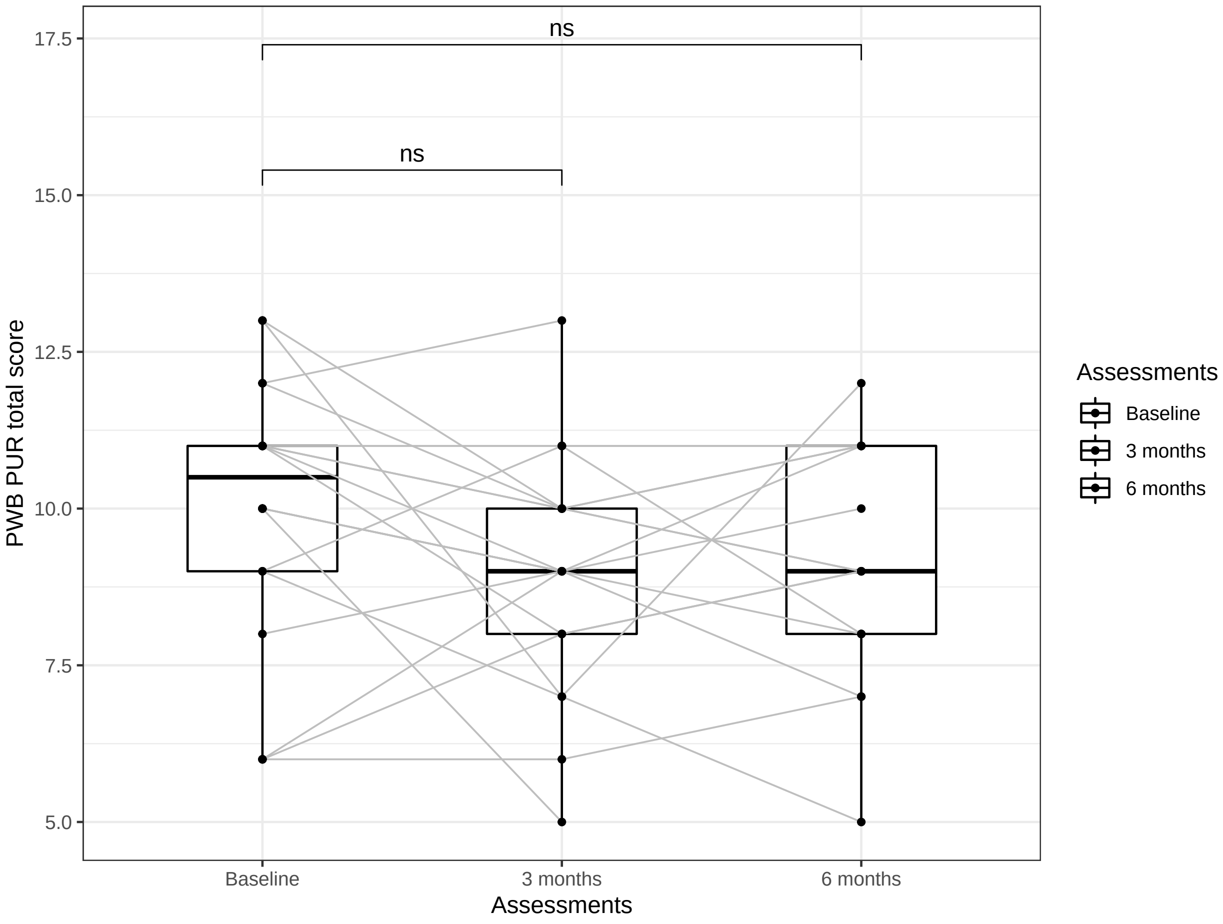


Supplementary Figure 6:
Distributions and individual trends of total mean scores of PWB Aim in Life (PWB PUR) at baseline (n=20), after 3 months (n=20), and after 6 months (n=16) of device use; (ns) not significant at Wilcoxon-Mann-Whitney test.

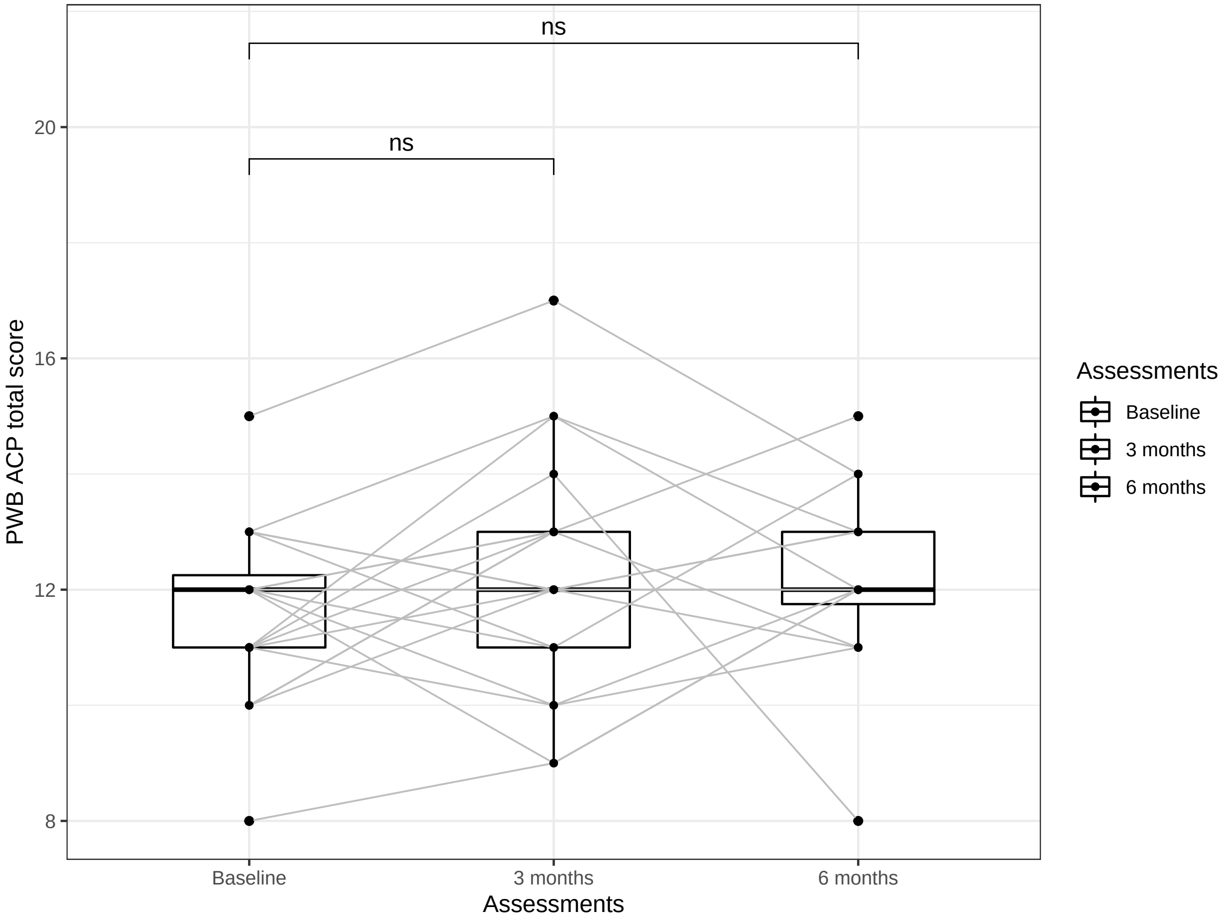


Supplementary Figure 7:
Distributions and individual trends of total mean scores of PWB Self-Acceptance (PWB ACP) at baseline (n=20), after 3 months (n=20), and after 6 months (n=16) of device use; (ns) not significant at Wilcoxon-Mann-Whitney test.
